# Pharmacokinetic Compatibility Study of Lidocaine with EXPAREL in Yucatan Miniature Pigs

**DOI:** 10.5402/2011/582351

**Published:** 2011-12-27

**Authors:** Brigitte M. Richard, Douglas E. Rickert, Dannette Doolittle, Amy Mize, Jason Liu, Charles F. Lawson

**Affiliations:** ^1^Clinical Research & Drug Safety Assessment, Pacira Pharmaceuticals Inc., San Diego, CA 92121, USA; ^2^Raleigh, NC 27613, USA; ^3^ABC Laboratories, Columbia, MO 65201, USA; ^4^Sinclair Research Center, LLC, Auxvasse, MO 65231, USA

## Abstract

We explored the potential for EXPAREL to interact with lidocaine. Sixty (60) male Yucatan Swine were randomized into 20 groups (*N* = 3/group). EXPAREL (2 or 4 mg/kg) and/or lidocaine HCl solution 1% or 2% (with epinephrine 1 : 200,000) were injected subcutaneously along a 5 cm virtual incision line. The effects on the pharmacokinetics of bupivacaine and lidocaine were examined when 5, 10, 20, and 40 minutes had passed between administration of lidocaine and EXPAREL. Systemic exposure to lidocaine was increased (AUC_0−24 hr_ by 48%; *C*_max_ by 1,640%) when lidocaine (4 mg/kg) was followed 5 minutes later by EXPAREL (4 mg/kg) compared to lidocaine administered alone. Plasma bupivacaine was increased (AUC_0−24 hr_ by 50–95%; *C*_max_ by 67–1,000%) when lidocaine (4 mg/kg) was followed 5 or 10 minutes later by EXPAREL (4 mg/kg) compared to EXPAREL alone. While EXPAREL should not be admixed with lidocaine, this study shows that local administration of EXPAREL after at least 20 minutes following local administration of lidocaine did not increase the release of either drug.

## 1. Introduction

DepoFoam bupivacaine (EXPAREL, bupivacaine liposome injectable suspension) is for single-dose infiltration into the surgical site to produce postsurgical analgesia. Among new drug delivery systems, EXPAREL is promising because such formulation leads to a slow release of bupivacaine, therefore, allowing a longer duration of action and a slower uptake into the systemic circulation, avoiding high plasma (and tissue) concentrations [1]. 

Another DepoFoam product, DepoDur (morphine sulfate extended-release liposome injection), can exchange its drug load with lidocaine when coadministered [2], leading to changes in systemic exposure. We therefore explored this potential with EXPAREL in a clinically relevant minipig model mimicking wound infiltration.

Lidocaine with epinephrine is commonly used for wound infiltration prior to surgery [3, 4]. In clinical practice, the use of a lidocaine-bupivacaine mixture has the theoretical advantage of allowing a smaller dose of each agent than if they were used alone. Lidocaine is expected to provide faster onset of sensory blockade after local nerve block and may be administered in the same field in surgical patients. Epinephrine decreases bleeding in the area of injection by constricting blood vessels. As a result, the use of epinephrine as a vasoconstrictor agent is expected to maximize any potential local interaction by decreasing local clearance from the injection [5].

The combined additive toxicologic effects of lidocaine and bupivacaine on central nervous (CNS) and cardiovascular (CV) systems have been reported [6–11], but there is a paucity of data evaluating the potential for drug interaction, from a pharmacokinetic (PK) perspective.

The objective of the present study was to quantify the degree of drug interaction that may occur when EXPAREL and lido are infiltrated subcutaneously (sc) within 5, 10, 20, or 40 minutes of each other at clinically relevant doses and concentrations.

The protocol included in vivo conditions that simulate the sc infiltration in a wound. The skin of pigs has a layer of sc tissue, which is very similar to that of man. The skin morphology (tight adhesiveness of the skin to the underlying structures), compared to rat, rabbit, or dog where the skin is loose, and physiology makes the swine a preferred model for extrapolation to humans [12–14].

Due to the clinical relevance of the miniature swine model used in this study, the present data were expected to provide a relevant prediction of the potential local interaction of EXPAREL when administered with lidocaine in humans. Therefore, dosing recommendations in a clinical setting were proposed.

## 2. Materials and Methods

### 2.1. Materials

#### 2.1.1. Description of DepoFoam Technology

The DepoFoam drug delivery system is a proprietary, injectable technology that provides a sustained release of therapeutic compounds. The DepoFoam system consists of microscopic, polyhedral, lipid-based particles composed of numerous nonconcentric, aqueous chambers containing the drug in solution.

Each chamber in this multivesicular liposome is separated from adjacent chambers by lipid membranes [15].

#### 2.1.2. Test Article

DepoFoam bupivacaine (EXPAREL; bupivacaine extended-release liposome injection using multivesicular DepoFoam technology), 15 mg/mL (expressed as anhydrous bupivacaine base) was provided by Pacira Pharmaceuticals, Inc., San Diego, CA, USA. As needed, EXPAREL was diluted with 0.9% sodium chloride injection USP to achieve a target concentration of 7.5 mg/mL.

#### 2.1.3. Reference Product

Xylocaine-MPF, (1% or 2% lidocaine HCl with 1 : 200,000 epinephrine) was manufactured by AstraZeneca, Wilmington, DE, USA.

#### 2.1.4. Animals

Male naïve Yucatan Miniature Swine were received from Sinclair BioResources Auxvasse, MO, USA. The animals were 6 to 17 months of age on arrival, and 24–50 kg at the time of dosing.

### 2.2. Methods

#### 2.2.1. Study Protocol

The protocol was reviewed and approved by the Institutional Animal Care and Use Committee (IACUC) of Sinclair Research Center for compliance with regulations prior to study initiation. This study complies with all applicable sections of the Final Rules of the Animal Welfare Act regulation (9 CFR).

Animals were housed in individual, stainless-steel, metabolism cages throughout the study period. Animals were fed once a day with free access to water. During a 5-day acclimation period, the animals were trained to a sling device. The animals were not fasted overnight before dose administration.

Sixty (60) male Yucatan Swine were randomized into 20 groups (*N* = 3/group). EXPAREL and/or lidocaine solution 1% or 2% (with epinephrine 1 : 200,000) was administered sc at dose levels of either 1 or 2 mg/kg. Bupivacaine was incorporated into the sustained release material at a percent loading of 1.5% by weight and further diluted with 0.9% sterile sodium chloride to achieve the desired concentration. Different dose regimen combinations were studied. Control groups with equivalent dose and volume were used to allow useful comparison with the test formulation.

Groups 1 and 2 received EXPAREL alone (0.27 mL/kg). Groups 3 and 4 received lidocaine alone (0.2 mL/kg). All remaining groups received 0.2 mL/kg lidocaine followed by 0.27 mL/kg EXPAREL at a given interval of time later (5, 10, 20, or 40 minutes).

**Figure 1:**
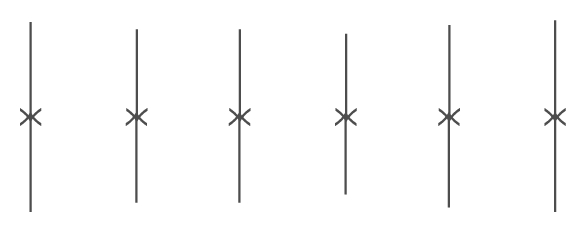
Diagram showing the dose equally distributed as 6 serial bolus injections along a 5 cm virtual incision line.

**Figure 2:**
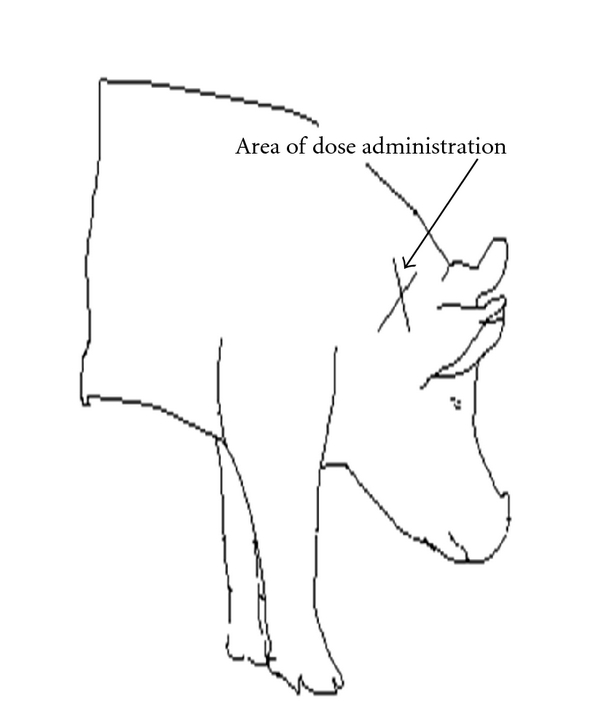
Diagram showing the approximate area where subcutaneous injections were administered on each animal (i.e., scapular region).

Since the recommended dose volume is approximately 2 mL/cm, the dose was equally distributed as 6 serial bolus injections along a 5 cm virtual incision line in the scapular region (Figures 1 and 2). Each injection was made perpendicular to the skin surface (i.e., directing the needle approximately 90 degrees), and each bolus was injected slowly (over 5 seconds) in the exact same spot as trailing injections along the line. The first injection (start of dosing) was performed beginning proximal to the line. The needle was redirected slightly more distal than the first pass of the needle, and the process was repeated a second time with another aliquot. All injections in a series were completed within 30–40 seconds. Blood samples were collected at predose, 5, 10, 15, 30 minutes, and 1, 2, 4, 6, 8, 12, 24, 48, 72, and 96 hours postdosing.

#### 2.2.2. Pharmacokinetic Assessment

Plasma concentrations were simultaneously analyzed by ABC Laboratories, Columbia, MO, USA, using a validated LC-MS/MS assay. The method is selective for the quantification of bupivacaine and lidocaine in K_3_EDTA plasma in the concentrations ranging from 0.25 to 100 ng/mL for each analyte. Depending on the time interval, between 5 and 40 minutes of plasma data for lidocaine were not collected since blood collection began after EXPAREL administration. This was taken into account when performing comparisons among groups. Systemic exposure parameters for lidocaine in the presence of bupivacaine represent minimal estimates only.

The PK parameters were evaluated by a noncompartmental model using WinNonlin, version 5.0 (Pharsight Corp., Mountain View, CA, USA). The PK parameters included maximum plasma concentration (*C*_max⁡_), time at which the *C*_max⁡_ occurred (*t*_max⁡_), and area under the plasma concentration-time data (AUC_0−*t*_). The appropriate group mean values and standard deviation (SD) were calculated from the individual data.

## 3. Results and Discussion

### 3.1. Results

**Figure 3:**
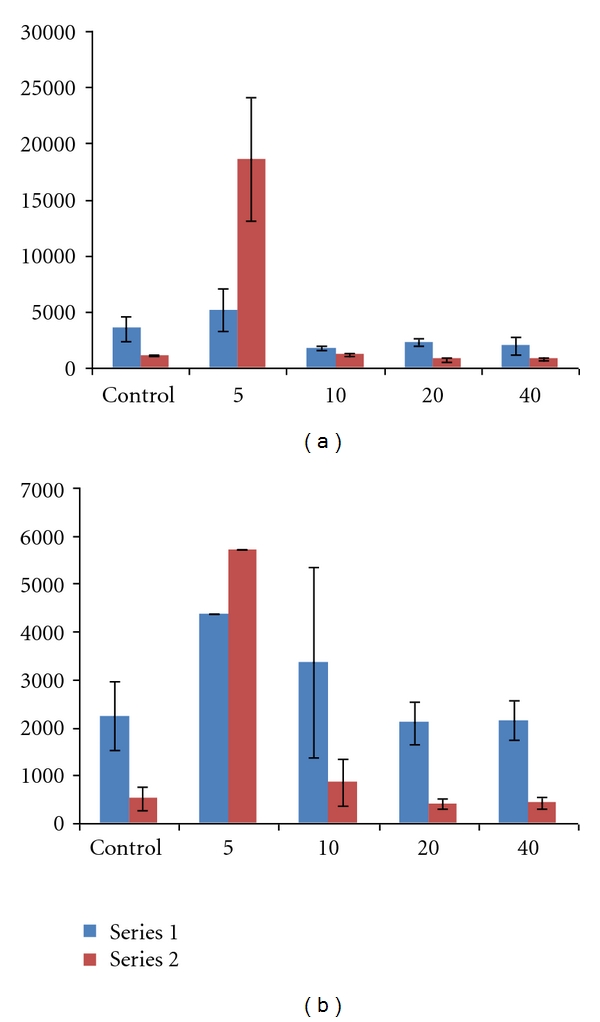
Mean systemic exposure parameters (±SD) for plasma lidocaine (a) and bupivacaine (b) after high (4 mg/kg) subcutaneous dose of lidocaine/epinephrine and EXPAREL (*N* = 3/group unless noted). Series 1: AUC_0−24 hr_ (ng/mL·hr). Series 2: *C*_max⁡_ (ng/mL). Notes: SD was not calculated for mean plasma bupivacaine concentrations at 5 minutes (*N* = 2) because one animal received 55% of the intended dose of EXPAREL; therefore, data from this animal were not used in calculation of means; individual values for AUC_0−24 hr_ = 2,980 and 5,770 ng/mL·hr and for *C*_max⁡_ = 3,690 and 7,770 ng/mL.

**Figure 4:**
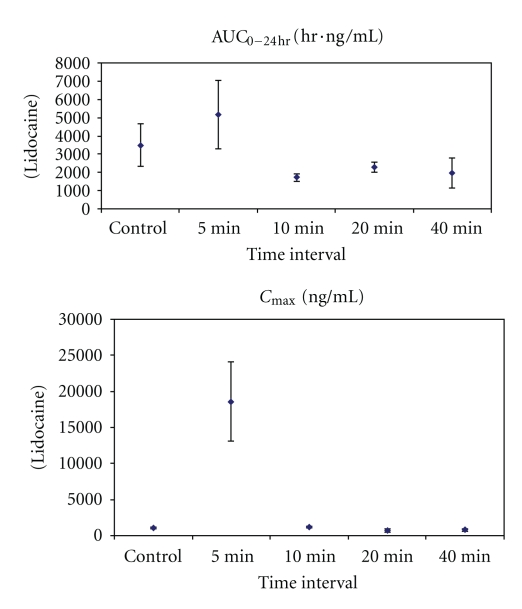
Mean systemic exposure parameters (±SD) for plasma lidocaine after high (4 mg/kg) subcutaneous dose of lidocaine/epinephrine and EXPAREL (*N* = 3/group).

**Figure 5:**
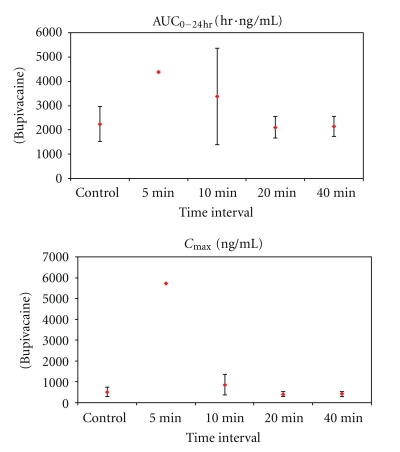
Systemic exposure parameters (±SD) for plasma bupivacaine after high (4 mg/kg) subcutaneous dose of lidocaine/epinephrine and EXPAREL (*N* = 3/group unless noted). Notes: SD was not calculated for bupivacaine at 5 minutes (*N* = 2, AUC = 2,980 and 5,770 ng/mL·h; *C*_max⁡_ = 3,690 and 7,770 ng/mL).

The PK results are shown in Tables 1 and 2 and Figures 3, 4, and 5. The most significant effects are highlighted below.

All doses were well tolerated in this model. Most differences in plasma parameters were clinically insignificant and were attributed to biological variations in individual responses.

Notably, there was no clinically meaningful difference in the mean systemic plasma exposure to either lidocaine or bupivacaine when EXPAREL was given 20 minutes or longer after lidocaine administration in studies using 4 mg/kg of lidocaine and EXPAREL.

While systemic exposure to lidocaine or bupivacaine was not increased when EXPAREL was administered 20 minutes or longer following lidocaine administration, when the interval was less than 20 minutes there were increases in systemic exposure. At the early timepoints (5 and 10 minutes), there was a marked difference in plasma lidocaine and/or bupivacaine (*C*_max⁡_, AUC_0−24 hr_) in animals receiving larger doses of more concentrated formulations.

When the high dose of lidocaine was followed by the high dose of EXPAREL (4 mg/kg each) by 5–10 minutes, the systemic exposure to lidocaine was increased over the exposure seen when the high dose of lidocaine was administered alone (Table 1) (*C*_max⁡_  1,070 ± 82.9 versus 18,600 ± 5, 472 ng/mL and AUC_0−24 hr_  872 ± 290 versus 1,290 ± 465 hr·ng/mL; *C*_max⁡_↑ 1,640%; AUC_0−24 hr_↑ 48%).

When the high dose of lidocaine was followed by the high dose of EXPAREL (4 mg/kg each) by 5–10 minutes, *C*_max⁡_ of bupivacaine (EXPAREL) was increased 67–1,000% (519 ± 230 versus 865 ± 488 and 5,730 ng/mL) and AUC_0−24 hr_ was 50–95% higher (2,240 ± 721 hr·ng/mL versus 4,370 and 3,370 ± 1, 980 hr·ng/mL) compared to when EXPAREL was administered alone (Table 2).

### 3.2. Discussion

Drug interactions are significant risk factors, especially, in surgical patients receiving multimodal analgesia. The magnitude of these interactions depends on a multitude of factors including dose volume, dose concentration, injection method, and timing/sequence of administration. 

In the present study, we evaluated the degree of drug interaction that may occur when EXPAREL is infiltrated 5, 10, 20, or 40 minutes after an injection of lidocaine at various clinically relevant dose ratios and drug concentrations.

We used a clinically relevant animal model with in vivo conditions that simulate the sc infiltration in a wound. A special injection infiltration procedure was used to facilitate close contact between lidocaine and bupivacaine while minimizing the risk of disruption of lipid vesicles and other disturbances of the injection area. This model was considered particularly relevant for this type of investigation due to skin similarities between the minipig and humans [12–14]. The doses selected in our studies were based on these recommendations. The recommended guideline in pigs is lidocaine given at 1–4 mg/kg maximum dose mixed with conventional bupivacaine 1-2 mg/kg before the incision is made [16].

When lidocaine is used for regional nerve blocks, plasma levels are usually 3,000–5,000 ng/mL; toxicities may be observed at 6,000 ng/mL, but more commonly occur once levels exceed 10,000 ng/mL. Therapeutic levels are usually 700–1,000 ng/mL while plasma threshold associated with systemic toxicity is 2,000 to 4,000 ng/mL for bupivacaine [17, 18]. As the toxicity of bupivacaine is known to be generally associated with its *C*_max⁡_, EXPAREL may be safer than unencapsulated bupivacaine solution at the same dose by allowing for a longer presence of bupivacaine at lower systemic peak levels.

There was no clinically meaningful difference in the mean systemic plasma exposure to either lidocaine or bupivacaine when EXPAREL was given 20 minutes or longer after lidocaine administration in studies using 4 mg/kg of lidocaine and EXPAREL. At the early timepoints (5 and 10 minutes), there was a marked difference in plasma lidocaine and/or bupivacaine (*C*_max⁡_, AUC_0−24 hr_) in animals receiving larger doses of more concentrated formulations.

It should be noted that the dosing procedure selected for this study maximizes the potential risk of drug interference. The injected concentrated formulations were in close proximity of each other, especially after being infiltrated in the same field and within a short time interval. The high-dose ratio (4 : 4) results highlight the possibility of a clinically relevant PK interference when EXPAREL is infiltrated within a shorter time interval following lidocaine solution. The involvement of epinephrine to vasoconstrict most likely allows greater interaction of the concentrated compounds at the injection sites.

It is not yet clear which precise mechanism(s) of action are involved in this model. The physiological mechanism is based in part upon the higher affinity of lidocaine to the DepoFoam. It is likely that the local interaction is influenced by diluting/mixing effects of the exposed sites in the presence of both formulations. It is also possible that, at shorter time intervals, increasing local blood flow accelerates systemic absorption of the injected formulation(s) since the vascular effects of lidocaine and bupivacaine are dynamic and dependent on concentration and time [19–22].

In summary, there was no increase in either drug when the administration of EXPAREL was delayed by 20 or 40 minutes after the injection of the lidocaine solution. While EXPAREL should not be admixed with lidocaine, administration of EXPAREL after at least 20 minutes following local administration of lidocaine did not increase the release of either drug. All other lidocaine/EXPAREL drug interaction effects were not considered clinically meaningful.

## 4. Conclusions

Based on the preclinical data, we conclude that local administration of EXPAREL after at least 20 minutes following local administration of lidocaine did not increase systemic exposure of either drug.

## Figures and Tables

**Table 1:** Pharmacokinetic parameters for lidocaine after administration of EXPAREL and lidocaine/epinephrine solution (4 : 4 dose ratio).

Time interval (min)^a^	Parameters for plasma lidocaine	
	(% change from lidocaine control)	
	AUC_0−24 hr_ (hr·ng/mL)	*C*_max⁡_ (ng/mL)
0	3,490 ± 1,160	1, 070 ± 82.9
5	5, 170 ± 1,860 (+48%)	18, 600 ± 5,472 (+1,640%)
10	1, 720 ± 202 (−51%)	1, 200 ± 104 (+12%)
20	2, 290 ± 269 (−34%)	723 ± 206 (−32%)
40	1, 980 ± 827 (−76%)	774 ± 127 (−28%)

^a^ *N* = 3 per group. Please refer to Section 2.2 for experimental details.

**Table 2:** Pharmacokinetic parameters for bupivacaine after administration of EXPAREL and lidocaine/epinephrine solution (4 : 4 dose ratio).

Time interval (min)^a^	Parameters for plasma bupivacaine	
	(% change from bupivacaine control)	
	AUC_0−24 hr_ (hr·ng/mL)	*C*_max⁡_ (ng/mL)
0	2,240 ± 721	519 ± 230
5^b^	4,370 (+95%) (2,980–5,770)	5,730 (+1,000%) (3,690–7,770)
10	3,370 ± 1,980 (+50%)	865 ± 488 (+67%)
20	2,100 ± 445 (−6%)	412 ± 113 (−21%)
40	2,150 ± 410 (−4%)	422 ± 119 (−19%)

^a^ *N* = 3 per group (unless noted); ^b^ *N* = 2; one animal received 55% of the intended dose of EXPAREL. Data from this animal were not used in calculation of means.

## Abbreviations

EXPAREL: Bupivacaine liposome injectable suspension using multivesicular DepoFoam technology
Bupi: Bupivacaine
CNS: Central nervous system
CV: Cardiovascular
Lido: Lidocaine
MPF: Methyl paraben free
PK: Pharmacokinetics
Sc: Subcutaneous(ly)
SD: Standard deviation.

## References

[1] Angst MS, Drover DR (2006). Pharmacology of drugs formulated with DepoFoam^®^: a sustained release drug delivery system for parenteral administration using multivesicular liposome technology. *Clinical Pharmacokinetics*.

[2] DepoDur^®^ (morphine sulfate extended- release liposome injection). http://www.drugs.com/pro/depodur.html.

[3] Duocaine^TM^ USPI (lidocaine HCl—bupivacaine HCl injection) 1% / 0.375%. http://www.drugs.com/pro/duocaine.html.

[4] Cuvillon P, Nouvellon E, Ripart J (2009). A comparison of the pharmacodynamics and pharmacokinetics of bupivacaine, ropivacaine (with epinephrine) and their equal volume mixtures with lidocaine used for femoral and sciatic nerve blocks: a double-blind randomized study. *Anesthesia and Analgesia*.

[5] Bernards CM, Kopacz DJ (1999). Effect of epinephrine on lidocaine clearance in vivo: a microdialysis study in humans. *Anesthesiology*.

[6] Krikava I, Jarkovský J, Stourac P, Nováková M, Sevcík P (2010). The effects of lidocaine on bupivacaine-induced cardiotoxicity in the isolated rat heart. *Physiological Research*.

[7] Simon L, Kariya N, Pelle-Lancien E, Mazoit JX (2002). Bupivacaine-induced QRS prolongation is enhanced by lidocaine and by phenytoin in rabbit hearts. *Anesthesia and Analgesia*.

[8] Mets B, Janicki PK, James MF, Erskine R, Sasman B (1992). Lidocaine and bupivacaine cardiorespiratory toxicity is additive: a study in rats. *Anesthesia and Analgesia*.

[9] Mazoit JX, Orhant EE, Boico O, Kantelip JP, Samii K (1993). Myocardial uptake of bupivacaine: I. Pharmacokinetics and pharmacodynamics of lidocaine and bupivacaine in the isolated perfused rabbit heart. *Anesthesia and Analgesia*.

[10] Fujita Y, Endoh S, Yasukawa T, Sari A (1998). Lidocaine increases the ventricular fibrillation threshold during bupivacaine-induced cardiotoxicity in pigs. *British Journal of Anaesthesia*.

[11] Clarkson CW, Hondeghem LM (1985). Evidence for a specific receptor site for lidocaine, quinidine, and bupivacaine associated with cardiac sodium channels in guinea pig ventricular myocardium. *Circulation Research*.

[12] Monteiro-Riviere NA, Bristol DG, Manning TO, Rogers RA, Riviere JE (1990). Interspecies and interregional analysis of the comparative histologic thickness and laser Doppler blood flow measurements at five cutaneous sites in nine species. *Journal of Investigative Dermatology*.

[13] Perez R, Davis SC (2008). Relevance of animal models for wound healing. *Wounds*.

[14] Sullivan TP, Eaglstein WH, Davis SC, Mertz P (2001). The pig as a model for human wound healing. *Wound Repair and Regeneration*.

[15] http://www.pacira.com/depofoam-about.aspx.

[16] http://depts.washington.edu/compmed/veterinary/pdf/guidelinesforanalgesia2008-14-06-appendices.pdf.

[17] Scott DB (1975). Evaluation of the toxicity of local anaesthetic agents in man. *British Journal of Anaesthesia*.

[18] Tucker GT, Mather LE (1979). Clinical pharmacokinetics of local anaesthetics. *Clinical Pharmacokinetics*.

[19] Newton DJ, McLeod GA, Khan F, Belch JJF (2005). Vasoactive characteristics of bupivacaine and levobupivacaine with and without adjuvant epinephrine in peripheral human skin. *British Journal of Anaesthesia*.

[20] Guinard JP, Carpenter RL, Morell RC (1992). Effect of local anesthetic concentration on capillary blood flow in human skin. *Regional Anesthesia*.

[21] Johns RA, DiFazio CA, Longnecker DE (1985). Lidocaine constricts or dilates rat arterioles in a dose-dependent manner. *Anesthesiology*.

[22] Johns RA, Seyde WC, DiFazio CA, Longnecker DE (1986). Dose-dependent effects of bupivacaine on rat muscle arterioles. *Anesthesiology*.

